# Impact of health-related behavioral factors on participation in a cervical cancer screening program: the lifelines population-based cohort

**DOI:** 10.1186/s12889-023-17293-0

**Published:** 2023-11-30

**Authors:** Kelly M. Castañeda, Grigory Sidorenkov, Marian J. E. Mourits, Bert van der Vegt, Albert G. Siebers, Karin M. Vermeulen, Ed Schuuring, G. Bea A. Wisman, Geertruida H. de Bock

**Affiliations:** 1 Department of Epidemiology, University of Groningen, University Medical Center Groningen, 9700 RB Groningen, the Netherlands; 2 Department of Gynaecologic Oncology, Cancer Research Center Groningen, University Medical Center Groningen, University of Groningen, Hanzeplein 1, 9713 GZ Groningen, the Netherlands; 3 Department of Pathology & Medical Biology, University of Groningen, University Medical Center Groningen, 9700 RB Groningen, the Netherlands; 4 Dutch Nationwide Pathology Databank, PALGA, 3991 SZ Houten, the Netherlands

**Keywords:** Uterine cervical neoplasms, Early detection of cancer, Patient compliance, Lifestyle, Reproductive history

## Abstract

**Background:**

Regular participation in cervical cancer screening is critical to reducing mortality. Although certain sociodemographic factors are known to be associated with one-time participation in screening, little is known about other factors that could be related to regular participation. Therefore, this study evaluated the association between health-related behavioral factors and regular participation in cervical cancer screening.

**Methods:**

The Lifelines population-based cohort was linked to data for cervical cancer screening from the Dutch Nationwide Pathology Databank. We included women eligible for all four screening rounds between 2000 and 2019, classifying them as regular (4 attendances), irregular (1–3 attendances), and never participants. Multinomial logistic regression was performed to evaluate the association between behavioral factors and participation regularity, with adjustment made for sociodemographic factors.

**Results:**

Of the 48,325 included women, 55.9%, 35.1%, and 9% were regular, irregular, and never screening participants. After adjustment for sociodemographic factors, the likelihood of irregular or never screening participation was increased by smoking, obesity, marginal or inadequate sleep duration, alcohol consumption and low physical activity, while it was decreased by hormonal contraception use.

**Conclusion:**

An association exists between unhealthy behavioral factors and never or irregular participation in cervical cancer screening.

**Supplementary Information:**

The online version contains supplementary material available at 10.1186/s12889-023-17293-0.

## Background

Organized screening programs in developed countries have triggered decreases in cervical cancer mortality, yet the disease remains one of the leading causes of cancer death [1, 2]. Although participation in cervical cancer screening may be associated with a reduced burden of disease [3], it requires a minimum participation level of 70% to be effective. Unfortunately, many European countries are yet to attain these levels [4, 5], and it is unclear how we can influence willingness to participate [3]. While various sociodemographic factors are known to be associated with non-participation in cervical cancer screening [6–8], studies of other screening programs have uncovered associations with health-related behavioral factors, such as smoking, alcohol consumption, higher body mass index (BMI), and oral contraception [9, 10]. For example, a healthier lifestyle score has been positively correlated with attendance at colonoscopy [11], while current or former oral contraception users seem more likely to attend multiple rounds of breast cancer screening. Regarding the role of such factors on participation in cervical cancer screening, studies have produced inconsistent results about the roles of smoking, alcohol consumption, physical activity, and reproductive factors [7, 8, 12–15]. Moreover, such research has been limited to evaluations of single screening attendances and the use of self-reported questionnaires to define attendance.

This study aimed to evaluate the association of health-related behavioral factors with participation in cervical cancer screening, adjusted for sociodemographic factors known to affect screening participation.

## Methods

### Study design and data sources

This cross-sectional research is nested in the Lifelines cohort, a multidisciplinary, prospective, population-based study using a unique three-generation design to examine the health and health-related behaviors of 167,729 people living in the north of the Netherlands. Lifelines employs a broad range of investigative procedures to assess the biomedical, sociodemographic, behavioral, physical, and psychological factors contributing to the health and disease of the general population, focusing on multi-morbidity and complex genetics [16–18]. Between 2007 and 2019, Lifelines conducted two in-person assessments and three online follow-up questionnaires. A third in-person assessment is currently ongoing. However, these assessments and follow-up times do not completely match the period evaluated for the screening rounds evaluated. Therefore, for the current study, we retrieved data on the most recent sociodemographic, reproductive, and lifestyle factors available from the Lifelines cohort and linked them to data from the Dutch Nationwide Pathology Databank (PALGA) for 2000–2020 to determine participation in cervical cancer screening.

### Setting: Dutch cervical cancer screening

In the Netherlands, primary screening for cervical cancer changed from cytology-based to high-risk human papillomavirus (hrHPV)-based testing in 2017 [19]. Before the change, women aged 30–60 years were invited every 5 years to undergo primary screening by cytology testing [19]. Since the change, women aged 30, 35, 40, 50, and 60 years have been invited to undergo primary hrHPV testing [20], with women aged 45, 55, and 65 years only invited if they had a hrHPV positive result or missed the last round of screening [20]. However, all women were tested in the first round of the hrHPV-based program (2017–2021) because their hrHPV statuses at ages 40, 50, and 60 years had not yet been established [20].

### Population

To address regular participation in cervical cancer screening, we included only women from the Lifelines cohort who were eligible for all the four cervical cancer screening rounds between 2000 and 2019 (i.e., born between 1955 and 1974) [21]. As age is the main factor to invite women for screening in the Netherlands, the birth year was used to define the eligibility year for each screening round (e.g. A woman who was born in 1970 is eligible for her first screening in 2000 when she turns 30, and in 2005 when she turns 35, and so on) [6]. Women were excluded if they had undergone hysterectomy (based on self-report in the Lifelines questionnaire before 2000 and their PALGA records thereafter) or if they died before screening (based on Lifelines questionnaires).

### Outcome

Data on participation in the cervical cancer screening were retrieved from PALGA records. Four screening rounds were evaluated: 2000–2004, 2005–2009, 2010–2014, and 2015–2019. In each screening round, a woman was considered participant when she had a primary screening test recorded within 36 months of the start of the eligibility year (except for women eligible in 2019, when we allowed a maximum time of 24 months). Otherwise she was considered non-participant [22]. Participation regularity was defined as follows: “regular” if women attended all four screening rounds, “irregular” if they attended one to three screening rounds, and “never” if we found no record of screening in any of the four rounds. Analyses on a second definition of regularity were also performed and are presented in the supplementary data.

### Exposures and confounders

All the exposure and confounders were retrieved from lifelines study. To ensure the use of the most recent Lifelines data, we only included data from the last questionnaire or assessment with the variables of interest; if missing, we used the next most recent questionnaire. The following health-related behavioral factors were used as the main exposures: smoking habits, alcohol consumption, Lifelines Diet Score (LLDS), BMI, physical activity, television (TV) watching (as a proxy for sedentarism), sleep duration, hormonal contraception use, number of children, and age first childbirth. In addition, we used country of birth/ethnicity, educational level, income, and marital status as confounders that have known associations with participation in cervical cancer screening.

Smoking status was categorized as never, former, and current. Never smokers answered “no” to the question “Have you ever smoked for as long as 1 year?” Former smokers had to report being smokers for ≥ 1 year or having stopped for at least 1 month before questioning. Current smokers answered “yes” to the question “Do you smoke now, or have you smoked in the last month?” [23].

Alcohol consumption was calculated by dividing the average number of alcohol glasses consumed per drinking day by the number of drinking days per month. It was then categorized as high (> 1.5 drinks per day), light to moderate (> 0 and ≤ 1.5 drinks per day on average), or none [24].

We calculated the LLDS from a food frequency questionnaire, considering the relative intakes of different food groups with known positive (e.g., vegetables) or negative (e.g., red or processed meat) health effects on a scale from 0 (lowest diet quality) to 48 (highest diet quality) [23]. The LLDS was then categorized as low (2–23), middle (24–28), and high (29–46) based on minimum and maximum scores of 2 and 46, respectively.

BMI was grouped into underweight (< 18.5 kg/m^2^), normal weight (18.5–24.9 kg/m^2^), overweight (25–29.9 kg/m^2^), and obese (≥ 30 kg/m^2^) categories [25].

Physical activity was evaluated by the Short Questionnaire to Assess Health-enhancing Physical Activity (SQUASH), although we only considered moderate-to-vigorous physical activity (MVPA) related to commuting and leisure time [23]. Based on the physical activity guideline set out by the Dutch Health Council, we categorized MVPA as low (<150 min/week), medium (150–299 min/week), and high (≥ 300 min/week). We then based sedentary behavior on the number of hours watching TV per day [26], categorized as low (≤ 2 h/day), medium (3–4 h/day), and high (≥ 5 h/day).

Total self-reported sleep per day was categorized according to the recommendations of the American National Sleep Foundation into adequate (7–9 h), marginal (6 or 10), and inadequate (< 6 or > 10 h).

For hormonal contraception, respondents could answer “yes” or “no” to the question “Have you ever used hormonal contraception?”.

We primarily used the child’s year of birth, as reported by mothers at the baseline and follow-up questionnaires, to estimate the number of births during the study. When this was absent, we used responses to the baseline question “how many children do you have?” All women who still had missing data were assumed to have no children if at least one questionnaire response indicated no pregnancies. Women were then grouped by the number of children (0, 1–2, and ≥ 3). The year of birth of the oldest child was used to estimate the age of the first child, and the mother’s age at this birth (≤ 26 years, 27–30 years, and ≥ 31 years) was estimated as the difference between her birth year and that of her oldest child.

Sociodemographic confounders included country of birth/ethnicity, educational level, and income, as reported previously [22], with the inclusion of marital status categorized into three groups: no partner, relationship without cohabiting, and relationship with cohabiting (including marriage).

### Statistical analysis

Sociodemographic and behavioral factors are presented by participation regularity, using the chi-squared test for linear trend to estimate the association between each exposure/confounder and the outcome. To evaluate the association of behavioral factors with participation regularity, we performed univariate analysis by multinomial logistic regression for all participants. Missingness was treated as an additional category for each variable in the univariable model and addressed by multiple imputation in the multivariable model. The multivariable model included all statistically significant variables and presented in a forest plot. Since the rate of missing data was slightly higher among never participants compared to regular and irregular participants, we conducted a sensitivity analysis by running two additional multivariate models. One used missingness as an additional category for each variable and the other included only participants with complete data for each variable in the model. Odds ratios (ORs) are reported with their 95% confidence intervals (95%CIs). All analyses were conducted using IBM SPSS Version 25.0 (IBM Corp., Armonk, NY, USA).

Due to the small number of missing values for BMI, they were not reported to protect the confidentiality of the participants.

## Results

Of the 48,325 women included from the Lifelines cohort, 27,018 (55.9%), 16,960 (35.1%), and 4,347 (9.0%) were regular, irregular, and never participants. Table 1 shows that from all included women, most women were born in the Netherlands (96%), educated to middle/higher levels (71%), had middle/higher incomes (65%), and cohabited (73%). Only education level showed no association with regular screening. Table 2 presents the health-related behavioral factors by regularity in cervical cancer screening.

**Table 1:** Sociodemographic characteristics by regularity in cervical cancer screening

Characteristics	Regular	Irregular	Never	P value*
	**n (%)**	**n (%)**	**n (%)**	
Total, *n* = 48,325	27,018 (55.9)	16,960 (35.1)	4,347 (9.0)	
*Year of birth*				
1955–1964	12,933 (47.9)	6,769 (39.9)	2,079 (47.8)	< 0.001
1965–1974	14,085 (52.1)	10,191 (60.1)	2,668 (52.2)	
*Country of birth / Ethnicity*				
The Netherlands	26,368 (97.6)	16,013 (94.4)	4,036 (92.8)	< 0.001
Other country	610 (2.3)	909 (5.4)	294 (6.8)	
Missing	40 (0.1)	38(0.2)	17 (0.4)	
*Educational level*				
Low	7,574 (28.0)	4,561 (26.9)	1,384 (31.8)	0.165
Middle	11,898 (44.0)	7,089 (41.8)	1,760 (40.5)	
High	7,350 (27.2)	5,142 (30.3)	1,149 (26.4)	
Missing	196 (0.7)	168 (1.0)	54 (1.2)	
*Income*				
Low	3,080 (11.4)	2,360 (13.9)	678 (15.6)	< 0.001
Medium	6,238 (23.1)	3,846 (22.7)	1,084 (24.9)	
High	12,562 (46.5)	7,508 (44.3)	1,717 (39.5)	
Unknown	5,138 (19.0)	3,246 (19.1)	868 (20.0)	
*Marital status*				
Relationship with cohabiting	21,976 (81.3)	12,781(75.4)	3,172 (73.0)	< 0.001
Relationship with no cohabiting	712 (2.6)	526 (3.1)	118 (2.7)	
No partner	2,664 (9.9)	2,123 (12.5)	705 (16.2)	
Missing	1,666 (6.2)	1,530 (9.0)	352 (8.1)	

*P*-values are based on chi-square for linear trend test

**Table 2:** Behavioral factors by regularity in cervical cancer screening

Behavioral factors	Regular	Irregular	Never	P value*
	**n (%)**	**n (%)**	**n (%)**	
*Smoking habits*				
Never smoker	12,317 (45.6)	7,330 (43.2)	1,804 (41.5)	< 0.001
Former smoker	10,131 (37.5)	5,962 (35.2)	1,514 (34.8)	
Current smoker	4,089 (15.1)	3,095 (18.2)	852 (19.6)	
Missing	481 (1.8)	573 (3.4)	177 (4.1)	
*Alcohol consumption*				
No	6,628 (24.5)	4,411 (26.0)	1,314 (30.2)	< 0.001
Light/moderate	18,223 (67.4)	10,685 (63.0)	2,503 (57.6)	
High	1,350 (5.0)	911 (5.4)	216 (5.0)	
Missing	817 (3.0)	953 (5.6)	314 (7.2)	
*Diet quality (LLDS)*				
Low	8,824 (32.7)	5,527 (32.6)	1,373 (31.6)	< 0.001
Middle	7,267 (26.9)	4,266 (25.2)	1,062 (24.4)	
High	6,663 (24.7)	4,035 (23.8)	967 (22.2)	
Missing	4,264 (15.8)	3,132 (18.5)	945 (21.7)	
*BMI*				
Underweight	211 (0.8)	129 (0.8)	37 (0.9)	< 0.001
Normal weight	11,993 (44.4)	7,261 (42.8)	1,771 (40.7)	
Overweight	9,630 (35.6)	5,862 (34.6)	1,500 (34.5)	
Obesity	< 5,178 (< 19.2)	< 3,692 (< 21.8)	< 1,038 (< 23.9)	
*Physical activity* (*MVPA)*				
Low	8,127 (30.1)	5,397 (31.8)	1,423 (32.7)	0.154
Middle	5,705 (21.1)	3,287 (19.4)	784 (18.0)	
High	10,889 (40.3)	6,332 (37.3)	1,598 (36.8)	
Missing	2,297 (8.5)	1,944 (11.5)	542 (12.5)	
*TV watching*				
Low	16,590 (61.4)	10,374 (61.2)	2,465 (56.7)	< 0.001
Middle	8,635 (32.0)	5,168 (30.5)	1,408 (32.4)	
High	1,342 (5.0)	931 (5.5)	307 (7.1)	
Missing	451 (1.7)	487 (2.9)	167 (3.8)	
*Sleep duration*				
Adequate	23,360 (86.5)	14,075 |(83.0)	3,468 (79.8)	< 0.001
Marginally too short/long	2,780 (10.3)	2,019 (11.9)	581 (13.4)	
Inadequate (too short/long)	441 (1.6)	384 (2.3)	137 (3.2)	
Missing	437 (1.6)	482 (2.8)	161 (3.7)	
*Hormonal contraception*				
No	1,286 (4.8)	1,050 (6.2)	369 (8.5)	0.939
Yes	22,035 (81.6)	13,451 (79.3)	3,191 (73.4)	
Missing	3,697 (13.7)	2,459 (14.5)	787 (18.1)	
*Number of children*				
0	3,041 (11.3)	2,163 (12.8)	857 (19.7)	< 0.001
1–2	15,447 (57.2)	9,401 (55.4)	2,228 (51.3)	
≥ 3	7,958 (29.5)	4,906 (28.9)	1,100 (25.3)	
Missing	572 (2.1)	490 (2.9)	162 (3.7)	
*Age at first child*				
≤ 26	8,623 (31.9)	4,940 (29.1)	1,412 (32.5)	< 0.001
27–30	8,443 (31.2)	4,914 (29.0)	1,009 (23.2)	
≥ 31	6,262 (23.2)	4,307 (25.4)	846 (19.5)	
No children	3,041 (11.3)	2,163 (12.8)	857 (19.7)	
Missing	649 (2.4)	636 (3.8)	223 (5.1)	

Missing are not reported for BMI to protect the confidentiality of the participants

*P*-values are based on chi-square for linear trend test

**Fig. 1:**
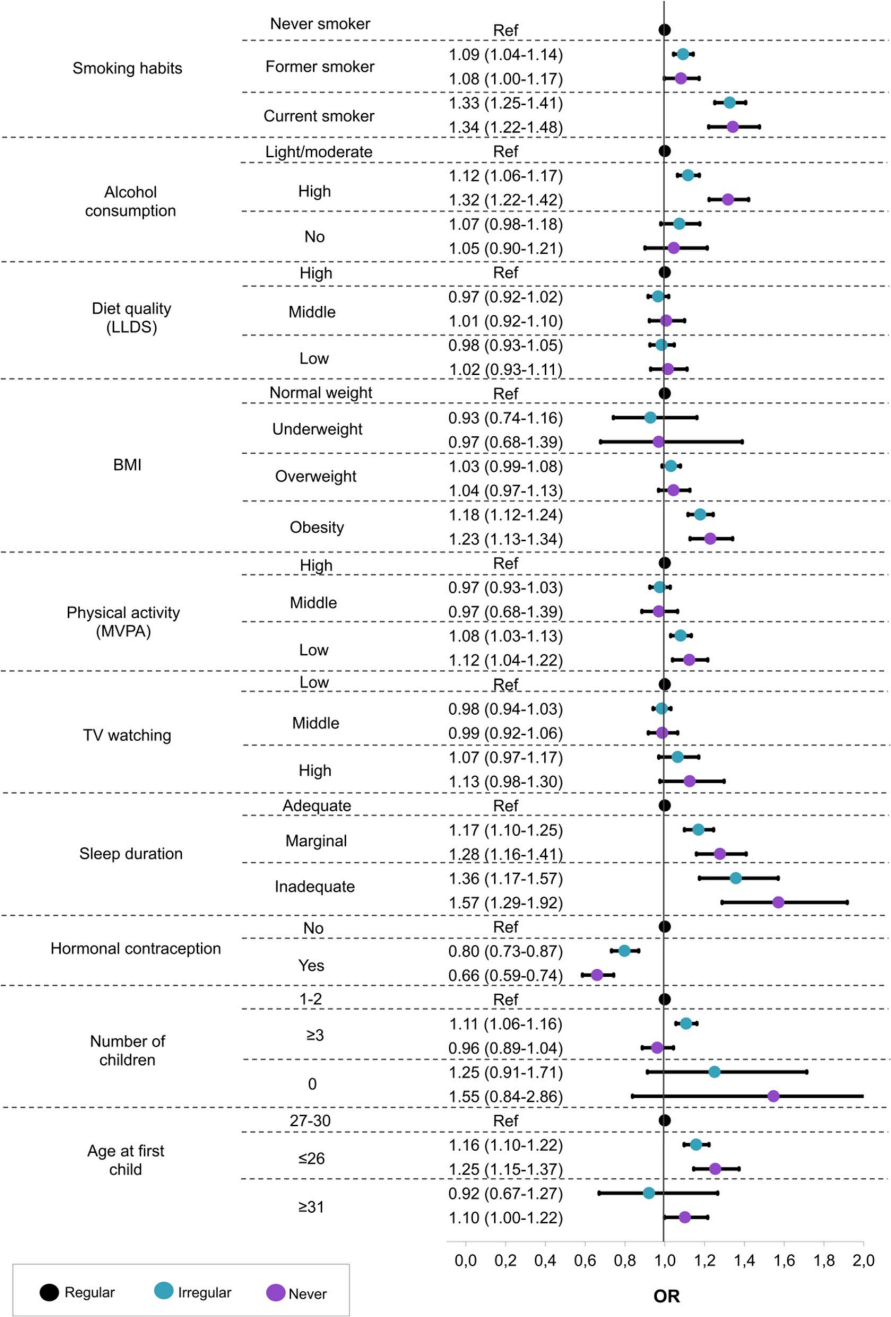
Association between lifestyle factors and regularity in the cervical cancer screening participation: a multinomial logistic regression (adjusted model). Imputed data. Model: smoking habits + alcohol consumption + LLDS + BMI + physical activity + TV watching + Sleep duration + hormonal contraception + number of children + age at first child + year of birth + country of birth/ethnicity + education + income + marital status

As shown in Supplementary Table 1, univariate analysis revealed significant associations between all health-related behavioral factors and participation regularity. As such, they could be included in the multivariable analysis (Fig. 1). After adjustment for sociodemographic factors, we found that smoking habits, alcohol consumption, BMI, physical activity, sleep duration, hormonal contraception, and age at the birth of the first child were independently associated with the regularity of screening participation. Compared with never smokers, we found that former and current smokers were more likely to participate irregularly or never. Similarly, women with high alcohol consumption, obesity, and low levels of MVPA were more likely to be irregular or never participants than their peers with light/moderate alcohol consumption, normal BMI, and high MVPA. Marginal or inadequate sleep was associated with a higher odds of irregular or never participation than adequate sleep. Additionally, we found a lower odds of irregular or never participation with any history of hormonal contraception use when compared with no history of use. Having three or more children increased the likelihood of irregular participation compared with having fewer children; however, this was not significant in those who never participated. Women aged 26 years or younger when they had children were more likely to be irregular or never participants than older women. LLDS and TV watching time were not associated with participation regularity.

In the sensitivity analyses (Supplementary Table 2), using imputed data alone, complete cases and missingness as additional categories only changed our findings for alcohol consumption. Performing the two extra models revealed that women who did not consume alcohol were more likely to be irregular or never participants, whereas no association existed for women with high alcohol consumption. The use of imputed data produced conflicting results.

When using the second definition for regularity, the overall results remained consistent. However, the trends became more evident when we defined regularity strictly as those who attended all four times (Supplementary Tables 3–8).

## Discussion

In this large cohort spanning 20 years in the north of the Netherlands, only 56% of eligible women participated regularly and a further 9% never participated in the Dutch cervical cancer screening program. After adjustment for well-known sociodemographic factors associated with participation, we found several health-related behaviors associated with an increased odds of irregular or never participation, including current or former smoking, no alcohol consumption, obesity, low physical activity, and childbirth before age 26 years. By contrast, current or ever hormonal contraceptive use was associated with a reduced odds of being irregular or never participants.

Among women in the Lifelines cohort eligible for the four cervical cancer screening rounds evaluated, our finding that 56% participated regularly is consistent with reported participation rates of 56% to 63% during this period [27]. Given that participation levels remained stable over this period, these figures could reflect accurate engagement levels. Also consistent with our finding of a 9% never participation rate, another study using data from PALGA revealed that around 10% of women have never participated in Dutch cervical cancer screening [28].

Screening must be regular to maximize the chance of detecting target lesions, yet we lack data on the key behavioral factors that affect participation [29, 30]. Our results agree with most existing studies of one-time participation, showing a higher odds of non-participation among women with any smoking history and low physical activity levels [8, 13, 15, 31]. These findings support the hypothesis that people with healthier lifestyles are more self-aware of their health and are more likely to comply with official advice about health prevention [8, 10, 32]. Also consistent with earlier reports, obese women were less likely to participate in cervical cancer screening due to weight-related barriers (e.g., embarrassment) [8, 15, 33].

The findings concerning alcohol consumption differed when using complete cases, imputed data, and missing data as an extra category, indicating that missingness was not at random. Therefore, the results for complete cases or missingness as an additional category are more suitable for interpretation (Supplementary Table 2). How alcohol consumption affects screening participation is unclear, with two published studies from Denmark and Norway reporting contradictory results for cervical cancer screening. The Danish study found no association with screening participation when measuring alcohol consumption by the number of units per week [15]. The Norwegian study, which considered alcohol consumption by type (i.e., beer, liquor, and wine), only found an association for wine drinking. Compared with women who drank wine one to three times per month, those who never drank wine had an increased odds of not participating in screening [12]. Our results support the Norwegian study in showing that women who never drank alcohol were more likely to be irregular or never screening participants. Given the broad acceptance of alcohol consumption in Dutch society (74% of Dutch women drink alcohol) [34], we hypothesize that the minority who do not drink have specific restrictions (e.g., disease, religion, medication) that affect both their alcohol consumption and participation in cervical cancer screening.

To the best of our knowledge, no other research has evaluated the association between sleep duration and screening participation. Our research also included sleep duration based on recent evidence suggesting that it has a critical impact on health [35], with irregular sleep being a modifiable factor that increases all-cause mortality [36]. Our results showed that women with inadequate and marginal sleep durations were more likely to be irregular or never participants in screening, lending further weight to the hypothesis that self-awareness and self-regulation of health status are reflected in the utilization of preventive health services [32].

We found a clear association between behavioral factors and screening participation in a native Dutch population. This association seemed related to self-awareness and self-regulation of health status, indicating that unhealthy behavioral factors might decrease the likelihood of screening participation. Thus, promoting healthy behaviors and self-awareness of health status might not only reduce cancer risk but also increase screening participation. Clinical trials have shown promise elsewhere [37, 38], indicating the opportunities to increase awareness and induce healthy lifestyle changes during the screening process [38]. Further studies using experimental designs are needed to evaluate these findings.

We included reproductive factors that might affect a woman’s willingness to participate in cervical cancer screening [39]. However, hormonal contraception was the only factor that reduced the odds of being an irregular or never participant in cervical cancer screening. This might reflect the necessary contact with a general practitioner (GP) for prescriptions [12]. For example, evidence from the Netherlands suggests that participation in cervical cancer screening increases significantly when involving GPs in the invitation process [6]. Since hormonal contraception use requires a prescription, for which women need to contact their GP at least once, this might provide an opportunity to give advice about the need for cervical cancer screening. Similarly, increased age is associated with planned pregnancy in the Netherlands [40], which is also associated with GP contact. This might explain why women who had their children before age 26 years had an increased likelihood of irregular or never participation in cervical cancer screening, because they will also have been less likely to have a planned pregnancy and GP contact.

This study benefited from being conducted in a large cohort nested within a population-based design [16, 17]. Indeed, the Lifelines cohort meant that we could include comprehensive data on many health-related behavioral factors [16, 17], which we could then link to data for cervical cancer screening from PALGA, a highly automated pathology databank with coverage close to 100% in the Netherlands [41]. In particular, the data from PALGA allowed for accurate information about participation regularity in cervical cancer screening. However, it is important to acknowledge the limitations of our study.

First, despite using the most recent Lifelines data for insights into current behavioral factors, the applicability of those data may ted by the possibility of fluctuations over the 20-year evaluation period. Still, a study in the Netherlands indicated that at the age of 25 years or older, behavioral factors remain stable over a 10-year period in at least 51% of individuals [42]. Therefore, we only expect a minor impact on our results. Second, we did not adjust for the influence of comorbidities in our analysis. Having comorbidities is known to be associated with lower participation in screening programs. However, cervical cancer screening invites women between 30 and 60, who have a relatively low risk of comorbidity [43]. Therefore, we do not expect major changes in our results if this this variables were included.

## Conclusion

This study offers the first evaluation of an association between health-related behavioral factors and participation regularity in cervical cancer screening with data spanning two decades and multiple rounds of screening. It shows that unhealthy behavioral factors are associated with irregular and never participation in cervical cancer screening, even after adjustment for sociodemographic factors. These factors may not only help characterize women who do not participate in screening but also inform the design of future prevention strategies. Promoting awareness of health statuses among women may eventually foster an increased willingness to participate in cervical cancer screening.

### Supplementary Information

**Additional file 1:** **Table S1 - Table S8.**

## Data Availability

The data used in this study are available through Lifelines biobank (www.lifelines.nl).

## Abbreviations

BMI: Body mass index
PALGA: Dutch Nationwide Pathology Databank
hrHPV: High-risk human papillomavirus
LLDS: Lifelines Diet Score
TV: Television
SQUASH: Short Questionnaire to Assess Health-enhancing Physical Activity
MVPA: Moderate-to-vigorous physical activity
