# Supplementary material for: Impact of health-related behavioral factors on participation in a cervical cancer screening program: the lifelines population-based cohort
Source: BMC Public Health. 2023 Nov 30;23:2376. doi: 10.1186/s12889-023-17293-0 (PMC10688458; doi:10.1186/s12889-023-17293-0)
Supplement: Supplementary file 1 — Additional file 1: Table S1 - Table S8. [file 12889_2023_17293_MOESM1_ESM.pdf]

Supplementary Table 1. Univariate analysis of the association between behavioral factors and participation in the Dutch cervical cancer screening program

**Behavioral factors**	**Participation***	
	**Irregular**	**Never**
Smoking habits		
Never smoker	Ref.	Ref.
Former smoker	0.99 (0.95–1.03)	1.02 (0.95–1.10)
Current smoker	1.27 (1.20–1.34)	1.42 (1.30–1.56)
Missing	2.00 (1.77–2.27)	2.51 (2.10–3.01)
Alcohol consumption		
Light/moderate	Ref.	Ref.
High	1.15 (1.06–1.26)	1.17 (1.00–1.35)
No	1.13 (1.08–1.19)	1.44 (1.34–1.55)
Missing	1.99 (1.81–2.19)	2.80 (2.44–3.21)
Diet quality (LLDS)		
High	Ref.	Ref.
Middle	0.94 (0.89–0.99)	0.94 (0.86–1.02)
Low	0.97 (0.92–1.02)	0.93 (0.85–1.02)
Missing	1.17 (1.01–1.24)	1.42 (1.30–1.56)
BMI		
Normal weight	Ref.	Ref.
Underweight	1.01 (0.81–1.26)	1.19 (0.83–1.69)
Overweight	1.01 (0.96–1.05)	1.06 (0.98–1.14)
Obesity	1.18 (1.12–1.24)	1.36 (1.25–1.48)
Missing	4.41 (1.72–11.26)	1.13 (0.13–9.38)
Physical activity (MVPA)		
High	Ref.	Ref.
Middle	0.99 (0.94–1.05)	0.94 (0.86–1.03)
Low	1.15 (1.09–1.20)	1.19 (1.11–1.29)
Missing	1.46 (1.36–1.56)	1.61 (1.44–1.79)
TV watching		
Low	Ref.	Ref.
Middle	0.96 (0.92–0.99)	1.10 (1.02–1.18)
High	1.11 (1.02–1.21)	1.54 (1.35–1.76)
Missing	1.73 (1.52–1.97)	2.50 (2.08–2.99)
Sleep duration		
Adequate	Ref.	Ref.
Marginally too short/long	1.83 (1.61–2.09)	1.41 (1.28–1.55)
Inadequate (too short/long)	1.45 (1.26–1.66)	2.09 (1.72–2.54)
Missing	1.21 (1.13–1.28)	2.48 (2.06–2.98)
Hormonal contraception		
No	Ref.	Ref.
Yes	0.75 (0.69–0.81)	0.51 (0.45–0.57)
Missing	0.82 (0.74–0.90)	0.74 (0.65–0.85)
Number of children		
1–2	Ref.	Ref.
≥3	1.01 (0.97–1.06)	0.96 (0.89–1.04)
0	1.17 (1.10–1.24)	1.95 (1.79–2.13)
Missing	1.41 (1.24–1.59)	0.96 (1.64–2.35)
Age at first child		
27–30	Ref.	Ref.
≤26	0.98 (0.94–1.03)	1.37 (1.26–1.49)
≥31	1.18 (1.12–1.25)	1.13 (1.03–1.25)
No children (0)	1.22 (1.15–1.31)	2.36 (2.14–2.61)
Missing	1.68 (1.51–1.89)	2.88 (2.44–3.39)

Univariate model using all women (n = 48,325), including missing in each variable as a category. Data are shown as odds ratios (ORs) and 95% confidence intervals (95% CIs).

* Comparison with regular participation

Supplementary Table 2. Multivariate analysis of the association between behavioral factors and participation in the Dutch cervical cancer screening program

Behavioral factors	All cases (n=48,325)		Complete cases (n=27,729)		Imputed data (n=48,325)	
	Participation*		Participation*		Participation*	
	Irregular	Never	Irregular	Never	Irregular	Never
Smoking habits						
Never smoker	Ref	Ref	Ref	Ref	Ref	Ref
Former smoker	1.10 (1.05–1.15)	1.09 (1.01–1.17)	1.08 (1.02–1.15)	1.08 (0.98–1.20)	1.09 (1.04–1.14)	1.08 (1.00–1.17)
Current smoker	1.32 (1.24–1.40)	1.33 (1.21–1.47)	1.23 (1.14–1.33)	1.32 (1.16–1.51)	1.33 (1.25–1.41)	1.34 (1.22–1.48)
Missing	1.11 (0.90–1.37)	1.71 (1.25–2.33)	NA	NA	NA	NA
Alcohol consumption						
Light/moderate	Ref	Ref	Ref	Ref	Ref	Ref
High	1.08 (0.98–1.18)	1.05 (0.90–1.22)	1.12 (1.00–1.26)	1.11 (0.91–1.35)	1.12 (1.06–1.17)	1.32 (1.22–1.42)
No	1.12 (1.07–1.17)	1.31 (1.22–1.42)	1.11 (1.04–1.18)	1.32 (1.19–1.47)	1.07 (0.98–1.18)	1.05 (0.90–1.21)
Missing	1.34 (1.14–1.57)	1.73 (1.38–2.17)	NA	NA	NA	NA
Diet quality (LLDS)						
High	Ref	Ref	Ref	Ref	Ref	Ref
Middle	0.97 (0.92–1.02)	0.98 (0.89–1.07)	0.98 (0.92–1.04)	1.00 (0.90–1.11)	0.97 (0.92–1.02)	1.01 (0.92–1.10)
Low	1.02 (0.97–1.08)	0.97 (0.89–1.07)	1.01 (0.95–1.08)	0.99 (0.89–1.11)	0.98 (0.93–1.05)	1.02 (0.93–1.11)
Missing	1.24 (1.08–1.43)	0.83 (0.65–1.06)	NA	NA	NA	NA
BMI						
Normal weight	Ref	Ref	Ref	Ref	Ref	Ref
Underweight	0.92 (0.74–1.15)	0.96 (0.67–1.38)	0.79 (0.57–1.10)	1.06 (0.65–1.71)	0.93 (0.74–1.16)	0.97 (0.68–1.39)
Overweight	1.03 (0.99–1.08)	1.05 (0.97–1.13)	1.02 (0.96–1.08)	1.05 (0.95–1.16)	1.03 (0.99–1.08)	1.04 (0.97–1.13)
Obesity	1.17 (1.11–1.23)	1.22 (1.12–1.33)	1.19 (1.11–1.28)	1.29 (1.15–1.46)	1.18 (1.12–1.24)	1.23 (1.13–1.34)
Missing	3.34 (1.29–8.66)	0.55 (0.07–4.69)	NA	NA	NA	NA
Physical activity (MVPA)						
High	Ref	Ref	Ref	Ref	Ref	Ref
Middle	0.97 (0.92–1.03)	0.97 (0.88–1.06)	0.96 (0.90–1.02)	1.00 (0.89–1.13)	0.97 (0.93–1.03)	0.97 (0.88–1.06)
Low	1.08 (1.03–1.13)	1.15 (1.06–1.24)	1.09 (1.03–1.16)	1.13 (1.02–1.25)	1.08 (1.03–1.13)	1.12 (1.04–1.22)
Missing	1.08 (1.00–1.18)	1.08 (0.94–1.25)	NA	NA	NA	NA
TV watching						
Low	Ref	Ref	Ref	Ref	Ref	Ref
Middle	0.99 (0.94–1.03)	1.01 (0.94–1.09)	0.98 (0.93–1.04)	1.03 (0.93–1.13)	0.98 (0.94–1.03)	0.99 (0.92–1.06)
High	1.06 (0.97–1.16)	1.16 (1.01–1.33)	1.18 (1.04–1.33)	1.13 (0.93–1.38)	1.07 (0.97–1.17)	1.13 (0.98–1.30)
Missing	1.11 (0.79–1.56)	1.23 (0.71–2.12)	NA	NA	NA	NA
Sleep duration						
Adequate	Ref	Ref	Ref	Ref	Ref	Ref
Marginally too short/long	1.16 (1.09–1.24)	1.28 (1.16–1.42)	1.15 (1.06–1.25)	1.26 (1.10–1.44)	1.17 (1.10–1.25)	1.28 (1.16–1.41)

Inadequate (too short/long)	1.34 (1.16–1.54)	1.62 (1.33–1.98)	1.16 (0.94–1.43)	1.95 (1.48–2.56)	1.36 (1.17–1.57)	1.57 (1.29–1.92)
Missing	1.24 (0.88–1.76)	0.72 (.40–1.27)	NA	NA	NA	NA
Hormonal contraception						
No	Ref	Ref	Ref	Ref	Ref	Ref
Yes	0.75 (0.69–0.82)	0.59 (0.52–0.67)	0.75 (0.67–0.83)	0.56 (0.48–0.65)	0.80 (0.73–0.87)	0.66 (0.59–0.74)
Missing	0.62 (0.52–0.73)	0.88 (0.67–1.15)	NA	NA	NA	NA
Number of children						
1–2	Ref	Ref	Ref	Ref	Ref	Ref
≥3	1.11 (1.06–1.16)	0.96 (0.89–1.05)	1.06 (0.99–1.13)	1.91 (1.66–2.20)	1.11 (1.06–1.16)	0.96 (0.89–1.04)
0	1.14 (1.06–1.22)	2.01 (1.81–2.24)	1.13 (1.03–1.23)	0.94 (0.84–1.05)	1.25 (0.91–1.71)	1.55 (0.84–2.86)
Missing	1.13 (0.78–1.64)	0.79 (0.49–1.30)	NA	NA	NA	NA
Age at first child						
27–30	Ref	Ref	Ref	Ref	Ref	Ref
≤26	0.99 (0.93–1.04)	1.25 (1.14–1.37)	0.99 (0.93–1.07)	1.27 (1.12–1.43)	1.16 (1.10–1.22)	1.25 (1.15–1.37)
≥31	1.16 (1.10–1.22)	1.11 (1.00–1.22)	1.16 (1.09–1.25)	1.07 (0.94–1.22)	0.92 (0.67–1.27)	1.10 (1.00–1.22)
Missing	1.17 (0.82–1.66)	2.32 (1.46–3.69)	NA	NA	NA	NA

For the three analyses: All cases (including missing as a category), complete cases (Including only women without missings in all the variables), and imputed data (using multiple imputations for the missings), the model was the following: smoking habits + alcohol consumption + LLDS + BMI + physical activity +TV watching + Sleep duration + hormonal contraception + number of children + age at first child + year of birth +country of birth/ethnicity +education +income + marital status.

* Comparison with regular participation

Outcomes definitions

Definition 1 (*Supplementary Tables 3-5)*.

a. **Regular:** participate 4 and 3 times
b. **Irregular:** 2 times
c. **Once in screening:** 1 time
d. **Non participant:**0 times

Supplementary table 3. Sociodemographic characteristics by regularity in cervical cancer screening.

**Characteristics**	**Regular** n (%)	**Irregular** n (%)	**Once in screening** n (%)	**Never** n (%)	***P value****
Total=48,325	37,693	4,440	1,845	4,347	
Year of birth					
1955-1964	17,157 (45.5)	1,838 (41.4)	707 (38.3)	2,079 (47.8)	0.601
1965-1974	20,536 (54.5)	2,602 (58.6)	1,138 (61.7)	2,268 (52.2)	
Country of birth / Ethnicity					
The Netherlands	36,537 (96.9)	4,168 (93.9)	1,676 (90.8)	4,036 (92.8)	<0.01
Other country	<1,098 (<2.9)	<263 (<5.9)	<158 (<8.6)	<294 (<6.8)	
Educational level					
Low	10,391 (27.6)	1,233 (27.8)	511 (27.7)	1,384 (31.8)	0.015
Middle	16,425 (43.6)	1,828 (41.2)	734 (39.8)	1,760 (40.5)	
High	10,585 (28.1)	1,330 (30.0)	577 (31.3)	1,149 (26.4)	
Missing	292 (0.8)	49 (1.1)	23 (1.2)	54 (1.2)	
Income					
Low	4,497 (11.9)	657 (14.8)	286 (15.5)	678 (15.6)	<0.01
Medium	8,636 (22.9)	1,024 (23.1)	424 (23.0)	1,084 (24.9)	
High	17,437 (46.3)	1,895 (42.7)	738 (40.0)	1,717 (39.5)	
Unknown	7,123 (18.9)	864 (19.5)	397 (21.5)	868 (20.0)	
Marital status					
Relationship with cohabiting	30,258 (80.3)	3,227 (72.7)	1,272 (68.9)	3,172 (73.0)	<0.01
Relationship with no cohabiting	1,029 (2.7)	148 (3.3)	61 (3.3)	118 (2.7)	
No partner	3,862 (10.2)	637 (14.3)	288 (15.6)	705 (16.2)	
Missing	2,544 (6.7)	428 (9.6)	224 (12.1)	352 (8.1)	

** chi-square for linear trend*
Missing are not reported for Country of birth to protect the confidentiality of the participants.

Supplementary table 4. Behavioral factors by regularity in cervical cancer screening.

Behavioral factors	Regular<br>n (%)	Irregular<br>n (%)	Once in screening<br>n (%)	Never<br>n (%)	*P value**
Smoking habits					
Never smoker	16,997 (45.1)	1,910 (43.0)	740 (40.1)	1,804 (41.5)	<0.001
Former smoker	13,995 (37.1)	1,506 (33.9)	592 (32.1)	1,514 (34.8)	
Current smoker	5,912 (15.7)	856 (19.3)	416 (22.5)	852 (19.6)	
Missing	789 (2.1)	168 (3.8)	97 (5.3)	177 (4.1)	
Alcohol consumption					
No	9,340 (24.8)	1,145 (25.8)	554 (30.0)	1,314 (30.2)	<0.001
Light/moderate	25,107 (66.6)	2,767 (62.3)	1,034 (56.0)	2,503 (57.6)	
High	1,935 (5.1)	226 (5.1)	100 (5.4)	216 (5.0)	
Missing	1,311 (3.5)	302 (6.8)	157 (8.5)	314 (7.2)	
Diet quality (LLDS)					
Low	12,294 (32.6)	1,445 (32.5)	612 (33.2)	1,373 (31.6)	<0.001
Middle	10,032 (26.6)	1,098 (24.7)	403 (21.8)	1,062 (24.4)	
High	9,248 (24.5)	1,028 (23.2)	422 (22.9)	967 (22.2)	
Missing	6,119 (16.2)	869 (19.6)	408 (22.1)	945 (21.7)	
BMI					
Underweight	282 (0.7)	38 (0.9)	20 (1.1)	37 (0.9)	<0.001
Normal weight	16,636 (44.1)	1,841 (41.5)	778 (42.2)	1,771 (40.7)	
Overweight	13,376 (35.5)	1,520 (34.2)	596 (32.3)	1,500 (34.5)	
Obesity	<7,399 (<19.6)	<1,041 (<23.4)	<451 (<24.4)	<1,039 (<23.9)	
Physical activity (MVPA)					
Low	17,280 (45.8)	2,299 (51.8)	1,005 (54.5)	2,296 (52.8)	<0.001
Middle	9,171 (24.3)	941 (21.4)	394 (21.4)	925 (21.3)	
High	<11,205 (<29.7)	<1,195 (<26.9)	<446 (<24.2)	<1,125 (<25.9)	
TV watching					
Low	23,188 (61.5)	2,678 (60.3)	1,098 (59.5)	2,465 (56.7)	<0.001
Middle	11,942 (31.7)	1,320 (29.7)	541 (29.3)	1,408 (32.4)	
High	1,870 (5.0)	271 (6.1)	132 (7.2)	307 (7.1)	
Missing	693 (1.8)	171 (3.9)	74 (4.0)	167 (3.8)	
Sleep duration					
Adequate	32,359 (85.8)	3,610 (81.3)	1,466 (79.5)	3,468 (79.8)	<0.001
Marginally too short/long	3,991 (10.6)	569 (12.8)	239 (13.0)	581 (13.4)	
Inadequate (too short/long)	661 (1.8)	99 (2.2)	65 (3.5)	137 (3.2)	
Missing	682 (1.8)	162 (3.6)	75 (4.1)	161 (3.7)	
Hormonal contraception					
No	1,859 (4.9)	303 (6.8)	174 (9.4)	369 (8.5)	0.873
Yes	30,644 (81.3)	3,473 (78.2)	1,369 (74.2)	3,194 (73.4)	
Missing	5,190 (13.8)	664 (15.0)	302 (16.4)	787 (18.1)	
Number of children					
0	4,237 (11.2)	634 (14.3)	333 (18.0)	857 (19.7)	<0.001
1-2	21,535 (57.1)	2,413 (54.3)	900 (48.8)	2,228 (51.3)	
≥3	11,070 (29.4)	1,255 (28.3)	539 (29.2)	1,100 (25.3)	
Missing	851 (2.3)	138 (3.1)	73 (4.0)	162 (3.7)	
Age at first child					
≤26	11,749 (31.2)	1,276 (28.7)	538 (29.2)	1,412 (32.5)	<0.001
27-30	11,688 (31.0)	1,224 (27.6)	445 (24.1)	1,009 (23.2)	
≥31	9,023 (23.9)	1,116 (25.1)	430 (23.3)	846 (19.5)	
No children	4,237 (11.2)	634 (14.3)	333 (18.0)	857 (19.7)	
Missing	996 (2.6)	190 (4.3)	99 (5.7)	223 (5.1)	

**chi-square for linear trend*

Missing are not reported for BMI and physical activity to protect the confidentiality of the participants

Supplementary table 5. Association between behavioral factors and regularity in the cervical cancer screening participation. Multinomial logistic regression

Behavioral factors	Ref: Regular		
	Irregular	Once in screening	Never
Smoking habits			
Never smoker	Ref.	Ref.	Ref.
Former smoker	1.03 (0.95-1.11)	**1.15 (1.02-1.29)**	1.06 (0.98-1.38)
Current smoker	**1.27 (1.16-1.39)**	**1.66 (1.46-1.90)**	**1.25 (1.11-1.37)**
Missing	0.81 (0.60-1.08)	1.305 (0.86-1.97)	**1.51 (1.12-2.03)**
Alcohol consumption			
Light/moderate	Ref.	Ref.	Ref.
High	0.99 (0.84-1.13)	1.12 (0.90-1.39)	1.02 (0.88-1.18)
No	1.07 (0.99-1.15)	**1.36 (1.22-1.52)**	**1.28 (1.19-1.38)**
Missing	**1.48 (1.18-1.85)**	**1.58 (1.15-2.18)**	**1.62 (1.304-2.02)**
Diet quality (LLDS)			
High	Ref.	Ref.	Ref.
Middle	0.96 (0.88-1.05)	**0.86 (0.75-0.97)**	0.98 (0.90-1.07)
Low	0.99 (0.90-1.08)	0.99 (0.87-1.13)	0.97 (0.89-1.06)
Missing	**1.38 (1.12-1.68)**	**1.23 (0.91-1.66)**	0.81 (0.64-1.02)
BMI			
Normal weight	Ref.	Ref.	Ref.
Underweight	1.10 (0.78-1.55)	1.22 (0.77-1.95)	1.02 (0.72-1.46)
Overweight	1.05 (0.97-1.12)	0.98 (0.87-1.09)	1.03 (0.96-1.11)
Obesity	**1.24 (1.14-1.35)**	**1.22 (1.08-1.38)**	**1.18 (1.09-1.29)**
Missing	1.99 (0.64-6.18)	**4.89 (1.65-14.53)**	
Physical activity (MVPA)			
High	Ref.	Ref.	Ref.
Middle	0.96 (0.88-1.05)	1.09 (0.946-1.25)	1.03 (0.94-1.13)
Low	**1.12 (1.05-1.21)**	**1.24 (1.10-1.39)**	**1.19 (1.10-1.29)**
Missing	0.66 (0.24-1.83)	-	0.22 (0.03-1.70)
TV watching			
Low	Ref.	Ref.	Ref.
Middle	0.95 (0.89-1.03)	0.95 (0.85-1.06)	1.01 (0.94-1.08)
High	1.33 (0.99-1.30)	**1.23 (1.01-1.50)**	**1.16 (1.02-1.33)**
Missing	**1.92 (1.21-3.05)**	0.91 (0.42-1.96)	1.29 (0.76-2.19)
Sleep duration			
Adequate	Ref.	Ref.	Ref.
Marginally too short/long	**1.21 (1.09-1.33)**	**1.20 (1.04-1.39)**	**1.24 (1.13-1.37)**
Inadequate (too short/long)	1.17 (0.94-1.45)	**1.72 (1.32-2.25)**	**1.51 (1.25-1.83)**
Missing	0.95 (0.58-1.55)	1.25 (0.59-2.67)	0.65 (0.37-1.14)
Hormonal contraception			
No	Ref.	Ref.	Ref.
Yes	**0.73 (0.64-0.83)**	**0.53 (0.45-0.63)**	**0.62 (0.55-0.70)**
Missing	**0.52 (0.41-0.66)**	**0.47 (0.33-0.67)**	0.93 (0.72-1.21)
Number of children			
1-2	Ref.	Ref.	Ref.
≥3	**1.09 (1.01-1.18)**	**1.28 (1.14-1.44)**	0.95 (0.87-1.02)
0	**1.28 (1.14-1.44)**	**1.68 (1.43-1.98)**	**1.61 (1.45-1.79)**
Missing 3	1.45 (0.92-2.30)	1.36 (0.72-2.56)	0.86 (0.55-1.35)
Age at first child			
≤26	Ref.	Ref.	Ref.
27-30	1.00 (0.92-1.09)	0.90 (0.79-1.03)	**0.79 (0.73-0.87)**
≥31	**1.16 (1.06-1.28)**	1.12 (0.97-1.30)	**0.85 (0.77-0.94)**
Missing	0.93 (0.61-1.42)	1.17 (0.65-2.11)	**1.61 (1.05-2.46)**

Adjusted model: Smoking habits+ alcohol consumption + LLDS + BMI + Physical activity + TV watching + Sleeping duration + hormonal contraception + Number of children + age at the first children + year of birth +country of birth/ethnicity +education +income +marital status.

Definition 2 (*Supplementary Tables 6-8*).

a. **Regular:** participate 4 and 3 times
b. **Irregular:** 2 and 1 time
c. **Non participant:**0 times

Supplementary table 6. Sociodemographic characteristics by regularity in cervical cancer screening

**Characteristics**	**Regular** n (%)	**Irregular** n (%)	**Never** n (%)	***P value****
Total=48,325	37,693	6,285	4,347	
Year of birth				
1955-1964	17,157 (45.5)	2,545 (40.5)	2,079 (47.8)	0.481
1965-1974	20,536 (54.5)	3,740 (59.5)	2.268 (52.2)	
Country of birth / Ethnicity				
The Netherlands	36.537 (96.9)	5,844 (93.0)	4,036 (92.8)	<0.001
Other country	1,098 (2.9)	421 (6.7)	294 (6.8)	
Missing	58 (0.2)	20 (0.3)	17 (0.4)	
Educational level				
Low	10,391 (27.6)	1,744 (27.7)	1,384 (31.8)	0.022
Middle	16,425 (43.6)	2,562 (40.8)	1,760 (40.5)	
High	10,585 (28.1)	1,907 (30.3)	1,149 (26.4)	
Missing	292 (0.8)	72 (1.1)	54 (1.2)	
Income				
Low	4,497 (11.9)	934 (15.0)	678 (15.6)	<0.001
Medium	8,636 (22.9)	1,448 (23.0)	1,084(24.9)	
High	17,437 (46.3)	2,633 (41.9)	1,717 (39.5)	
Unknown	7,123 (18.9)	1,261 (20.1)	868 |(20.0)	
Marital status				
Relationship with cohabiting	30,258 (80.3)	4,499 (71.6)	3,172 (73.0)	<0.001
Relationship with no cohabiting	1,029 (2.7)	209 (3.3)	118 (2.7)	
No partner	3,862 (10.2)	925 (14.7)	705 (16.2)	
Missing	2,544 (6.7)	652 (10.4)	352 (8.1)	

chi-square for linear trend

Supplementary table 7. Behavioral factors by regularity in cervical cancer screening.

**Behavioral factors**	**Regular n (%)**	**Irregular n (%)**	**Never n (%)**	***P value****
Smoking habits				
Never smoker	16,997 (45.1)	2,650 (42.2)	1,804 (41.5)	
Former smoker	13,995 (37.1)	2,098 (33.4)	1,514 (34.8)	<0.001
Current smoker	5,912 (15.7)	1,272 (20.2)	852 (19.6)	
Missing	798 (2.1)	265 (4.2)	117 (4.1)	
Alcohol consumption				
No	9,340 (24.8)	1,699 (27.0)	1,314 (30.2)	
Light/moderate	25,107 (66.6)	3,801 (60.5)	2,503 (57.6)	<0.001
High	1,935 (5.1)	326 (5.2)	216 (5.0)	
Missing	1,311 (3.5)	459 (7.3)	314 (7.2)	
Diet quality (LLDS)				
Low	12,294 (32.6)	2,057 (32.7)	1,373 (31.6)	
Middle	10,032 (26.6)	1,501 (23.9)	1,062 (24.4)	<0.001
High	9,248 (24.5)	1,450 (23.1)	967 (22.2)	
Missing	6,119 (16.2)	1,277 (20.3)	945 (21.7)	
BMI				
Underweight	282 (0.7)	58 (0.9)	37 (0.9)	
Normal weight	16,635 (44.1)	2,619 (41.7)	1,771 (40.7)	<0.001
Overweight	13,376 (35.5)	2,116 (33.7)	1,500 (34.5)	
Obesity	<7,387 (<19.6)	<1,483 (<23.6)	<1,038 (<23.9)	
Physical activity (MVPA)				
Low	17,280 (45.8)	3,304 (52.6)	2,296 (52.8)	
Middle	9,171 (24.3)	1.335 (21.2)	925 (21.3)	<0.001
High	<11,205 (<29.7)	<1,641 (<26.1)	<1,125 (<25.9)	
TV watching				
Low	23,188 (61.5)	3,776 (60.1)	2,465 (56.7)	
Middle	11,942 (31.7)	1,861 (29.6)	1,408 (32.4)	<0.001
High	1,870 (5.0)	403 (6.4)	307 (7.1)	
Missing	693 (1.8)	245 (3.9)	167 (3.8)	
Sleep duration				
Adequate	32,359 (85.8)	5,076 (80.8)	3,468 (79.8)	
Marginally too short/long	3,991 (10.6)	808 (12.9)	581 (13.4)	<0.001
Inadequate (too short/long)	661 (1.8)	164 (2.6)	137 (3.2)	
Missing	682 (1.8)	237 (3.8)	161 (3.7)	
Hormonal contraception				
No	1,859 (4.9)	477 (7.6)	369 (8.5)	
Yes	30,644 (81.3)	4,842 (77.0)	3,191 (73.4)	0.851
Missing	5,190 (13.8)	966 (15.4)	787 (18.1)	
Number of children				
0	4,237 (11.2)	967 (15.4)	857 (19.7)	
1-2	21,535 (57.1)	3,313 (52.7)	2,228 (51.3)	<0.001
≥3	11,070 (29.4)	1,794 (28.5)	1,100 (25.3)	
Missing	851 (2.3)	211 (3.4)	162 (3.7)	
Age at first child				
≤26	11,749 (31.2)	1,814 (28.9)	1,412 (32.5)	
27-30	11,688 (31.0)	1,669 (26.6)	1,009 (23.2)	
≥31	9.023 (23.9)	1,546 (24.6)	846 (15.5)	<0.001
No children	4,237 (11.2)	967 (15.4)	857 (19.7)	
Missing	996 (2.6)	289 (4.6)	223 (5.1)	

**chi-square for linear trend*

Missing are not reported for BMI and physical activity to protect the confidentiality of the participants

Supplementary table 8. Association between behavioral factors and regularity in the cervical cancer screening participation. Multinomial logistic regression

**Behavioral factors**	**Ref: Regular**	
	**Irregular**	**Never**
Smoking habits		
Never smoker	Ref.	Ref.
Former smoker	1.06 (0.99-1.13)	1.06 (0.8-1.14)
Current smoker	**1.38 (1.27-1.49)**	**1.25 (1.14-1.37)**
Missing	0.94 (0.73-1.21)	**1.50 (1.11-2.03)**
Alcohol consumption		
Light/moderate	Ref.	Ref.
High	1.02 (0.90-1.15)	1.02 (0.88-1.18)
No	**1.15 (1.08-1.23)**	**1.28 (1.19-1.38)**
Missing	**1.50 (1.24-1.82)**	**1.62 (1.30-2.02)**
Diet quality (LLDS)		
High	Ref.	Ref.
Middle	0.93 (0.87-1.00)	0.98 (0.90-1.07)
Low	0.99 (0.92-1.07)	0.97 (0.89-1.06)
Missing	**1.33 (1.12-1.59)**	0.81 (0.64-1.02)
BMI		
Normal weight	Ref.	Ref.
Underweight	1.14 (0.85-1.52)	1.02 (0.72-1.45)
Overweight	1.02 (0.96-1.09)	1.03 (0.96-1.11)
Obesity	**1.23 (1.15-1.33)**	**1.18 (1.09-1.29)**
Missing	**2.88 (1.19-6.88)**	0.40 (0.51-3.07)
Physical activity (MVPA)		
High	Ref.	Ref.
Middle	0.99 (0.92-1.07)	1.03 (0.94-1.13)
Low	**1.15 (1.08-1.24)**	**1.19 (1.10-1.29)**
Missing	0.47 (0.17-1.28)	0.22 (0.03-1.71)
TV watching		
Low	Ref.	Ref.
Middle	0.95 (0.89-1.01)	1.01 (0.94-1.08)
High	**1.16 (1.03-1.31)**	**1.16 (1.02-1.33)**
Missing	**1.58 (1.04-2.40)**	1.29 (0.76-2.20)
Sleep duration		
Adequate	Ref.	Ref.
Marginally too short/long	**1.20 (1.11-1.31)**	**1.24 (1.13-1.34)**
Inadequate (too short/long)	**1.34 (1.12-1.60)**	**1.50 (1.24-1.82)**
Missing	1.01 (0.66-1.56)	0.65 (0.37-1.13)
Hormonal contraception		
No	Ref.	Ref.
Yes	**0.66 (0.59-0.73)**	**0.62 (0.55-0.70)**
Missing	**0.50 (0.40-0.61)**	0.93 (0.72-1.21)
Number of children		
1-2	Ref.	Ref.
≥3	**1.14 (1.07-1.22)**	0.95 (0.87-1.02)
0	**1.34 (1.27-1.53)**	**1.61 (1.45-1.79)**
Missing 3	1.44(0.97-2.15)	0.86 (0.55-1.36)
Age at first child		
≤26	Ref.	Ref.
27-30	0.97 (0.90-1.05)	**0.80 (0.73-0.87)**
≥31	**1.15 (1.06-1.25)**	**0.85 (0.77-0.94)**
Missing	0.98 (0.68-1.43)	**1.61 (1.05-2.46)**

Adjusted model: Smoking habits+ alcohol consumption + LLDS + BMI + Physical activity + TV watching + Sleeping duration + hormonal contraception + Number of children + age at the first children + year of birth +country of birth/ethnicity +education +income +marital status.
